# Characterisation of thermally treated beech and birch by means of quasi-static tests and ultrasonic waves

**DOI:** 10.1038/s41598-023-33054-w

**Published:** 2023-04-18

**Authors:** Hajir Al-musawi, Elisa Manni, Alexander Stadlmann, Bernhard Ungerer, Mojtaba Hassan Vand, Olaf Lahayne, Riccardo Nobile, Georg Baumann, Florian Feist, Ulrich Müller

**Affiliations:** 1 Department of Material Science and Process Engineering, Institute of Wood Technology and Renewable Materials, University of Natural Resources and Life Sciences, Vienna, Konrad Lorenz-Straße 24, 3430 Tulln an der Donau, Austria; 2 HYCOBILITY Engineering and Technologies GmbH, Stadiongasse 6-8/Top 28, 1010 Vienna, Austria; 3 Department of Wood Science and Technology, Mendel University, Zemědělská 3, 613 00 Brno, Czech Republic; 4 Institute for Mechanics of Materials and Structures, Vienna University of Technology (TU Wien), Lilienthalgasse 14/Arsenal, 1030 Vienna, Austria; 5 Department of Engineering for Innovation, University of Salento, Via per Monteroni, 73100 Lecce, Italy; 6 Vehicle Safety Institute, Graz University of Technology, Inffeldgasse 23/I, 8010 Graz, Austria

**Keywords:** Engineering, Materials science

## Abstract

Wood, being renewable and highly abundant material, with excellent high specific strength and stiffness, has received increasing attention to be used in high performance applications such as the structural element of a battery case in an electric vehicle. For a successful implementation of wood in the automotive sector, it is, therefore, crucial to understand the behaviour of wood during and after temperature exposure and in the event of fire with the presence/absence of oxygen. In this study, the mechanical properties of thermally modified and unmodified European beech and birch in air and nitrogen environments at six different treatment intensities were characterised using compression tests, tensile tests, shear tests and Poisson’s ratio tests. Further, the elastic properties of these wood species were quantified using the ultrasound measurements. The obtained strength and stiffness exhibited mild improvement upon moderate temperature treatment (200 °C), followed by a decrease at elevated temperature levels. This improvement was somewhat more pronounced under nitrogen treatment than under air treatment conditions. Nevertheless, a more noticeable decrease in the material performance was observed in beech compared to birch, occurring at earlier stages of modifications. This study confirms the tension–compression asymmetry of beech and birch where higher Young’s moduli were obtained from tensile than from compression tests for reference and thermally treated beech and birch. The shear moduli obtained from ultrasound for birch were comparable to those obtained from quasi-static tests, whereas there was an overestimation of approximately 11–59% for the shear modulus of beech compared to quasi-static tests. Poisson’s ratios from ultrasound tests corresponded well with those from quasi-static tests for untreated beech and birch, but not for thermally modified samples. The Saint-Venant model can satisfactorily predict the shear moduli of untreated and treated beech wood.

## Introduction

In times of global supply-chain issues and the need to decrease the dependence on fossil fuels, the use of renewable and locally available resources into technical applications becomes vital. Wood processed as an engineered timber product, being a renewable, all-year-round available resource with superior specific strength properties^1^, is a prominent candidate for substituting materials that require higher amounts of primary production energy^2,3^. A diverse field of applications highlights a rising number of wood-based solutions, from multi-storey buildings^4^ all the way to lightweight structural composites in the automotive sector^5^.

Regardless of the kind of structural element, safety requirements and technical standards beyond mechanical thresholds under standard climatic conditions have to be met. In this context, the risk of biological degradation^6^, dimensional instabilities owing to changes in the moisture content^7^ and thermal degradation under elevated temperatures^8^ are challenges to deal with when working with wood. To increase the biological resistance and dimensional stability, a number of modifications by means of chemical reactants have been investigated^9^. Among these, the thermal modification of wood is an effective procedure, which is already applied on an industrial scale^10^.

Throughout the past century several researchers analysed the impact of thermal treatments on softwood, finding that the ultimate strength levels for bending and tension decreased, while a balance or even a slight increase occurred for the parameters hardness and modulus of elasticity (*E*)^11–14^. These effects appeared to be more pronounced in softwood than in hardwood^15^. More recent studies^16^ have focused on the characterisation of hardwood, reporting no significant changes in strength, while the compressive modulus clearly increased between 15 and 38%. Borůvka et al.^17^ demonstrated that birch (*Betula pendula* R.) features a higher thermal resistance compared to otherwise superior beech wood, rendering it an appropriate option for thermal applications. For both soft and hardwood, however, it was shown that, beneath ignition temperatures, there is a negative correlation between *E* on the one hand and temperature or exposure time on the other^18^.

Besides the temperature, other atmospheric conditions, such as the moisture content and the amount of oxygen, determine the outcome of a thermal treatment. Rusche et al.^19^ compared closed with open treatment systems, hygrothermal with hydrothermal and anaerobic with treatments in air. It was observed that closed, hygrothermal treatments in air resulted in a more severe decline in mechanical properties. With respect to the impact on *E*, a mass loss of 8% to 10% was reported as a critical level above which the performance decreases.

While all these studies report critical thresholds and strength values under certain treatment conditions, the requirement for advanced technical applications for wood is to simulate and predict its mechanical behaviour under any constellation of feasible treatment conditions. Yet, the accuracy of numerical simulations depends heavily on using reliable properties of wood. Nevertheless, characterisation of thermally modified wood along the different orthotropic directions in quasi-static tests is laborious and time-consuming, especially when measuring shear strength and shear modulus, where difficulties arise in obtaining uniform stress distribution. Regarding these issues, ultrasonic tests, being fast and non-destructive, can offer advantages over the conventional destructive techniques for determining the nine elastic constants of wood. In this context, Gomez-Royuela et al.^20^ presented a study on characterising thermally treated beech wood (*Fagus sylvatica* L.) via quasi-static and dynamic measurements. For the latter, ultrasound tests were conducted, enabling measurements of all orthotropic elastic constants.

It is worth mentioning that the shear moduli of orthotropic materials can be approximately estimated from the values of Young’s moduli and Poisson’s ratios using, for instance, the well-known Saint-Venant principle^21^ cited in Dackermann et al.^22^ and in Nejati et al.^23^. The accuracy of this model for predicting the shear moduli of different wood species has not been intensively investigated. If proven to be accurate, such an approach can provide a convenient and time-efficient method to estimate the shear modulus of wood for design purposes.

These latest investigations aimed at the modelling of modified wood have not considered the impact of anaerobic treatments. As mentioned, wood is also expected to be used in the future for structural components in vehicle construction, such as the structural element of a battery case in an electric vehicle^24^. In this context, the frame of battery housing will be made of a wood-metal composite, in which wood is completely covered by a metal skin. In the event of internal or external fire, wood then undergoes thermal decomposition under oxygen deprivation. Therefore, understanding the behaviour of wood in the absence of oxygen is critical for the given application. Practically, it is not possible to measure the mechanical properties of wood at the moment of fire in a nitrogen atmosphere, but rather after temperature exposure.

The present work attempts to fill these knowledge gaps by providing quasi-static measurements based on tensile, compression and shear tests, as well as dynamic measurements based on ultrasound tests to determine the elastic constants of thermally treated beech and birch wood. To the best of the authors’ knowledge, no dataset has yet been published on the full set of elastic constants of thermally treated birch species. Besides untreated reference samples, test series at temperatures of 150, 200 and 250 °C for 10 and 30 min were conducted, including a comparison between modifications in an inert nitrogen atmosphere and in air. The effects of the modifications were observed after preconditioning the material in a standard climate chamber, similar to the real use conditions of the suggested application.

The hypothesis of this study is that birch possesses superior mechanical performance under thermal treatment compared to beech. Furthermore, it is hoped to confirm the reliability and effectiveness of ultrasound measurements in the context of thermal treatments on wood. The accuracy of the Saint-Venant principle in predicting shear modulus will also be assessed.

## Materials and methods

### Materials

**Figure 1:**
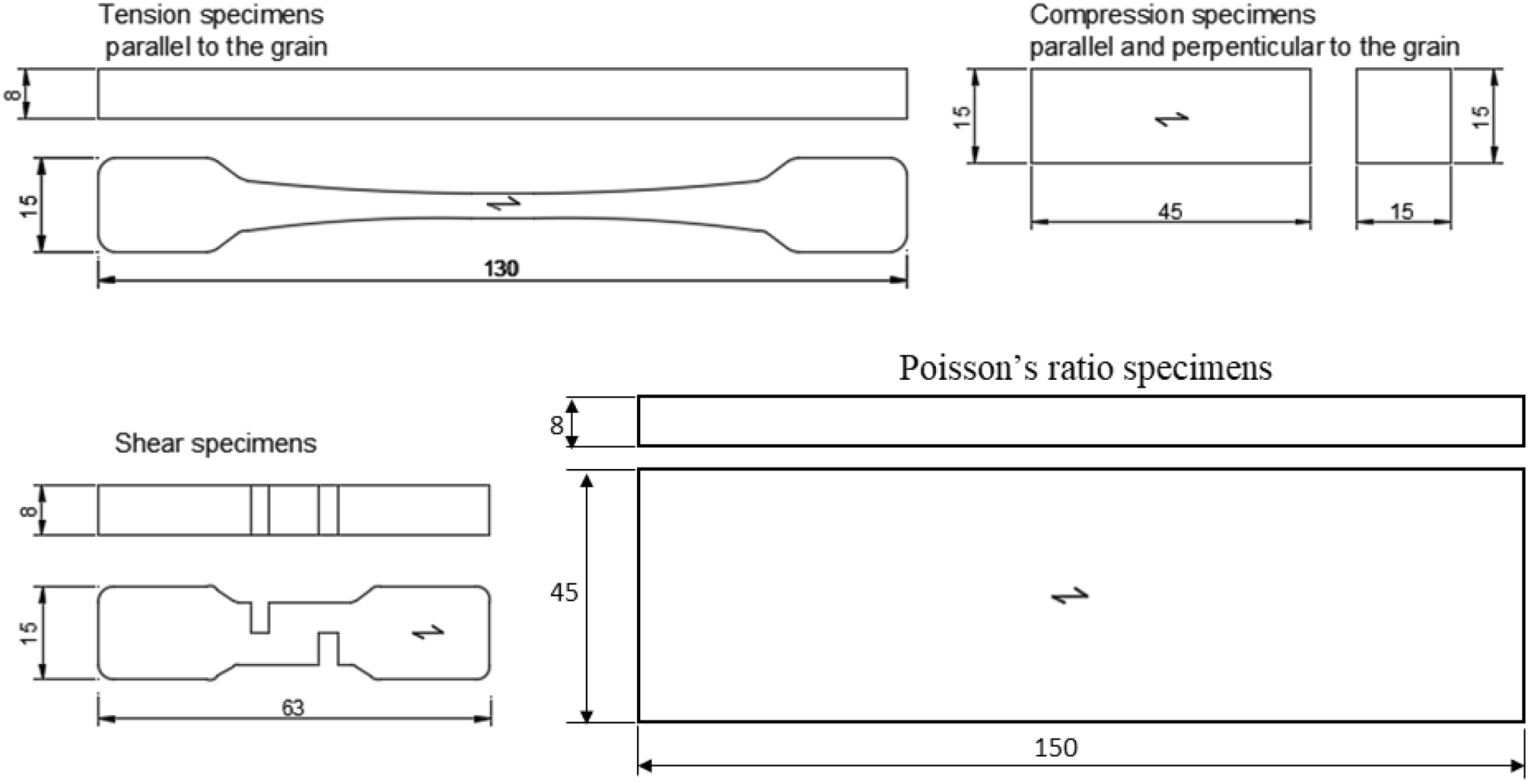
Specimen geometry for quasi-static tests (dimensions given are in mm).

Conventionally dried beech (*Fagus sylvatica*) and birch (*Betula pendula* Roth.) were sourced from the company Frischeis GmbH (Stockerau, Austria). The flawless wood material was split along axial direction with a wedge. The cleavage plane was planned flat with a jointer. Starting from this plane, the specimens were cut from the wood to ensure that the inclination of the fibres to the longitudinal axis deviated by less than 5° for all specimens. Specimens of different geometries were used for the quasi-static tests with a universal testing machine (see Fig. 1). A total of 556 specimens were tested for compression parallel and perpendicular to the fibre, Poisson’s ratio, tension and shear parallel to the fibre. Additionally, elastic constants in all wood anatomical directions were determined by means of ultrasonic tests on untreated and thermally treated (in air) specimens.

The geometries of tensile, compression and shear specimens were selected considering the dimensional constraints of the autoclave used for thermal treatment in an inert atmosphere, and their shape was scaled from the instructions of DIN 52188^25^, DIN 52185^26^ and DIN 52187^27^ standards, respectively. Meanwhile, the geometry of Poisson’s ratio specimens was chosen to minimise the measurement error by using a relatively wide specimen (45 mm).

### Thermal treatment

**Figure 2:**
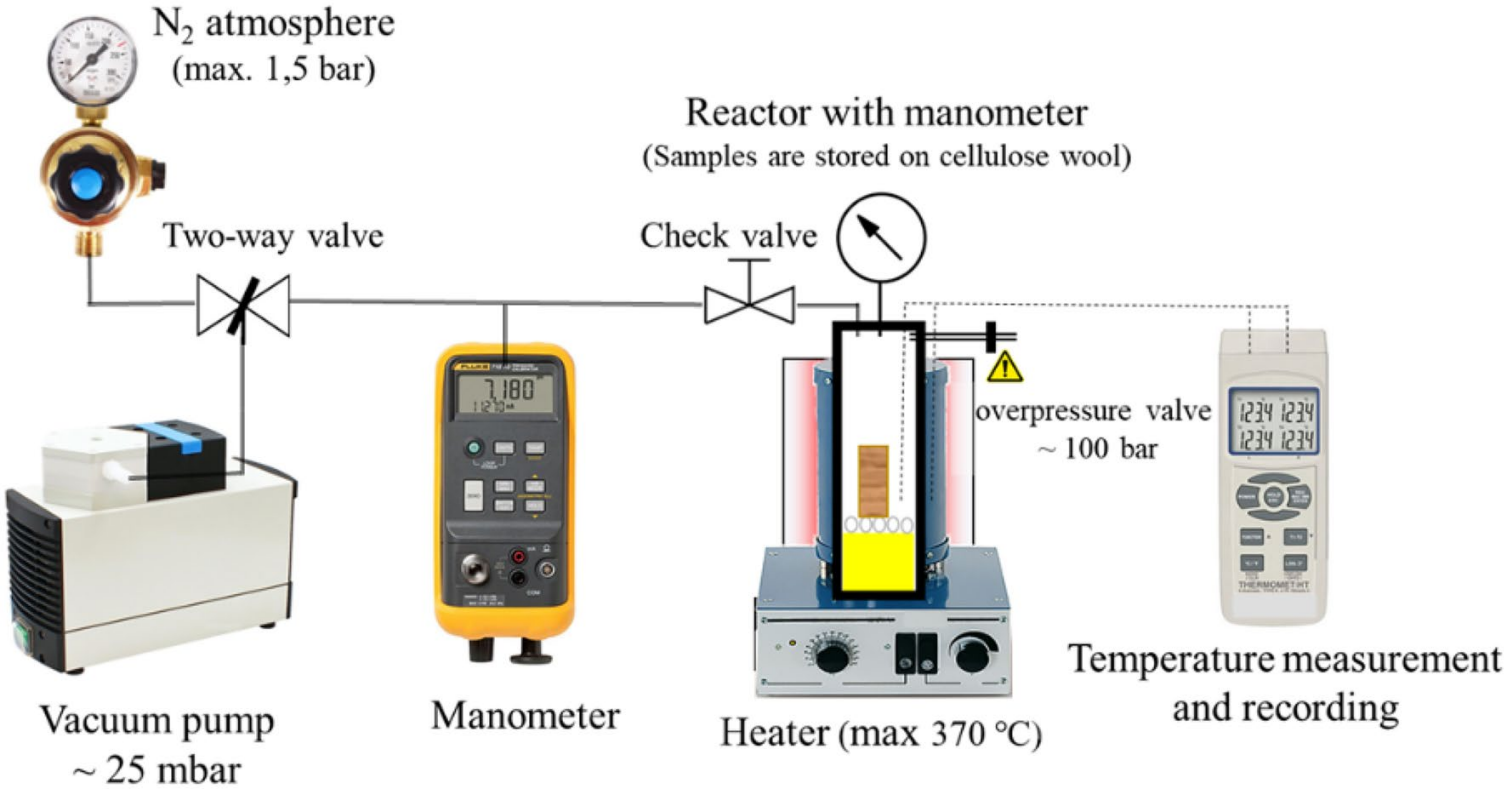
Thermal treatment in an inert atmosphere.

Before thermal treatment, all the samples were dried in an exicator at 60 °C for 24 h. This temperature was chosen to avoid possible oxidative reactions as well as avoiding any possible strength reductions with the usual 103 °C drying temperature^28^. Thermal treatment was carried out at three temperatures (150, 200, and 250 °C) for two treatment times (10 and 30 min) and under two different atmospheric conditions (oxygen and nitrogen). The equipment of nitrogen thermal modification is shown in Fig. 2. The settings combinations are summarised in Table 1.

**Table 1:** Thermal treatment conditions.

Atmosphere	Air		Nitrogen	
Time [min]	10	30	10	30
Temperature [°C]	150	150	150	150
	200	200	200	200
	250	250	250	250

The thermal treatment in air was performed in an oven with a recirculation fan, realising an open system treatment, while the treatment in an inert atmosphere was performed in a small autoclave. After the heating process, the specimens were reconditioned and stored in a climate chamber at 20 °C and a relative humidity of 65%. The investigated modified physical effects were mass loss (ML), density loss (DL) and change in moisture content. Their details are presented in the supplementary material.

### Quasi-static testing

The compression tests were conducted on a universal testing machine (Zwick/Roell Z100, Ulm, Germany). The force was recorded by the machine load cell with a capacity of 100 kN. Deformation of the specimens was based on the measurement of the crosshead displacement. For this purpose, the pressure plates of the universal testing machine were closed and the deformation under load was measured. For the determination of the true deformation of the specimens, the machine deformation was subtracted. The maximum force was used to calculate the compressive strength in the longitudinal direction, while the force corresponding to 0.2% permanent deformation was adopted for compressive strength in the radial direction. The Young’s modulus was evaluated as the slope of the elastic region in the stress–strain curve of the loading phase.

Meanwhile, tensile tests, shear tests and the Poisson’s ratio measurements were performed on a different universal machine (Zwick/Roell Z20, Ulm, Germany) with a load cell capacity of 20 kN. The contactless LaserXtens (Zwick/Roell, Ulm, Germany) was used to detect the longitudinal displacement with a resolution of 0.0001 mm^29^. The shear modulus *G*_*LR*_ (the first character of the subscript refers to the loading direction while the other character refers to the normal to the plane direction) was calculated as the ratio of τ_*LR*_ (the shear stress in the elastic range) to γ_*LR*_ (shear strain). γ_*LR*_ corresponds to recorded longitudinal elongation divided by the change in the length of the cross section in the radial direction.

The Poisson’s ratio (*ν*_*LR*_) was obtained from tensile tests, following the procedure presented by Kumpenza et al.^29^, which corresponds to the contraction strain in the radial direction (*ε*_*R*_) divided by the longitudinal direction (*ε*_*L*_).

The *G* values of thermally treated and untreated beech and birch were estimated using the Saint-Venant relation^21^, cited in Dackermann et al.^22^ and in Nejati et al.^23^, as given in Eq. 1.

1 $$\frac{1}{Gij}= \frac{1}{Ei}+ \frac{1+2vji}{Ej} \quad(\mathrm{wherei},\,\mathrm{j }=\mathrm{ L},\mathrm{ R},\mathrm{ T\,directions}).$$

### Ultrasound tests

**Figure 3:**
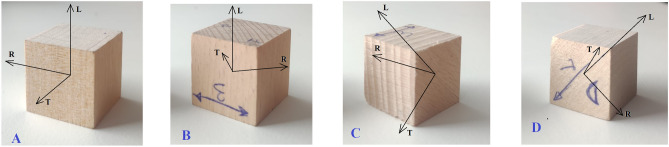
Specimens used for ultrasound measurements with different orientations.

A series of four cuboid specimens (20 × 20 × 20 mm) for each wood type and temperature level with orientations illustrated in Fig. 3 were used for ultrasound testing. The first of each four specimen is oriented along the main axes to derive the diagonal terms of the stiffness matrix, whereas the other specimens, oriented 45° to the planes RT, LT and LR, respectively, were required to obtain the off-diagonal components of the stiffness matrix by means of ultrasonic through-transmission method^30^. In this case, three longitudinal waves (*V*_*ii*_), six shear waves (*V*_*ij*_) and three quasi-shear waves at 45° (*V*_*ij*_/_*ij*_) were recorded. The stiffness parameters were computed from the mass density of the material and from the measured ultrasonic wave velocities. It should be remembered that, unlike quasi-static tests where elastic parameters are directly obtained as test results, ultrasonic tests provide the stiffness matrix [C], which needs to be inverted to calculate compliance matrix S, [S] = [C]^−1^. Then, the elastic parameters were computed as follows:

2 $$S_{ij} = \left| {\begin{array}{*{20}l} {\frac{1}{{E_{L} }}} \hfill & {\frac{{v_{RL} }}{{E_{R} }}} \hfill & {\frac{{v_{TL} }}{{E_{T} }}} \hfill & 0 \hfill & 0 \hfill & 0 \hfill \\ { - \frac{{v_{LR} }}{{E_{L} }}} \hfill & {\frac{1}{{E_{R} }}} \hfill & {\frac{{v_{TR} }}{{E_{T} }}} \hfill & 0 \hfill & 0 \hfill & 0 \hfill \\ { - \frac{{v_{LT} }}{{E_{L} }}} \hfill & { - \frac{{v_{RT} }}{{E_{R} }}} \hfill & {\frac{1}{{E_{T} }}} \hfill & 0 \hfill & 0 \hfill & 0 \hfill \\ 0 \hfill & 0 \hfill & 0 \hfill & {\frac{1}{{G_{RT} }}} \hfill & 0 \hfill & 0 \hfill \\ 0 \hfill & 0 \hfill & 0 \hfill & {} \hfill & {\frac{1}{{G_{TL} }}} \hfill & 0 \hfill \\ 0 \hfill & 0 \hfill & 0 \hfill & 0 \hfill & 0 \hfill & {\frac{1}{{G_{LR} }}} \hfill \\ \end{array} } \right|$$

where *E*_*L*_, *E*_*R*_, *E*_*T*_ are Young’s moduli, *G*_*LR*_, *G*_*TL*_, *G*_*RT*_ are the shear moduli and *ν*_*LR*_, *ν*_*RL*_, *ν*_*LT*_, *ν*_*TL*_, *ν*_*RT*_, *ν*_*TR*_ are the Poisson’s ratios. The first subscript letter refers to the propagation direction whereas the second letter refers to polarisation direction. Further details on the calculation procedure are reported elsewhere^30,31^.

Ultrasonic measurements were performed by means of a pulser-receiver (5077PR-Olympus NDT), a digital oscilloscope Wave Runner 62Xi-Lecroy. Ultrasonic longitudinal and transversal transducers with frequencies of 1000 kHz were employed which ensures that the wavelength (1–5 mm) was larger than heterogeneities within the wood meso-structure.

**Figure 4:**
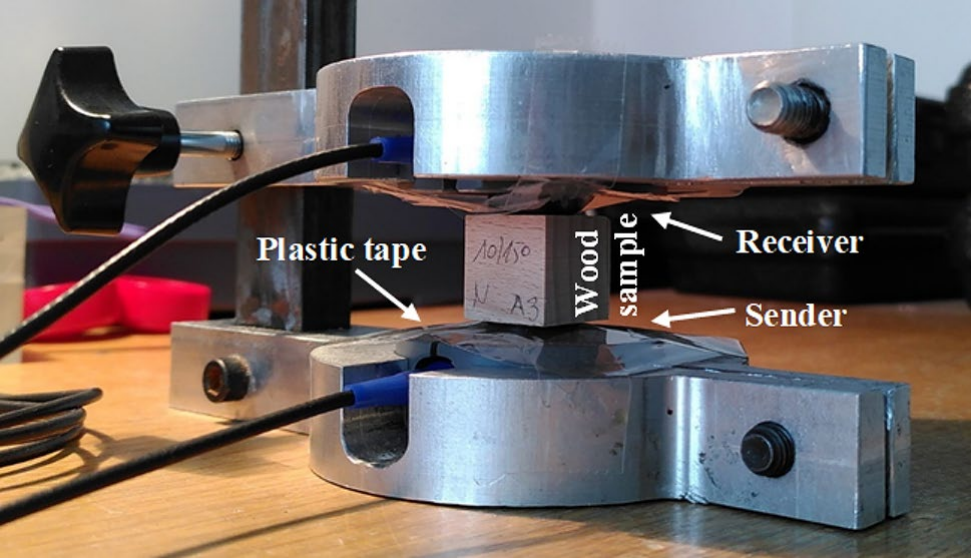
Ultrasound experimental setup.

The experimental setup is rather simple (Fig. 4). The wooden sample was placed between a sender and a receiver. To improve coupling as much as possible, a honey layer was applied between both transducers and the specimens. To stop the honey from penetrating the wood sample, a plastic tape was used to cover both honey layers. On an oscilloscope, the run times of the signals were measured, and out of these times and the dimensions of the samples, the sound velocities for the longitudinal and transversal waves were calculated.

### Statistical analysis

A one-way analysis of variance (ANOVA) was performed using the statistical software SPSS (IBM SPSS Statistics, version 27, IBM Corporation, New York, USA) with a 5% α-level of significance. Bonferroni post-hoc tests were conducted due to homogeneity of variances. When the conditions for homogeneity of variances were not met, Games-Howell post-hoc tests were performed instead. It has to be kept in mind that the parametric tests may not be entirely appropriate since the number of samples for numerous treatment conditions was fewer than six. Hence, the Kruskal Wallis Test was performed, and if shown to have a significant difference, then, Mann–Whitney U test was conducted to exactly identify which treatment conditions were statistically significant from their reference treatment. The results are given in the supplementary materials (Table S1).

## Quasi-static tests results and discussion

### Compressive, tensile and shear strength results

The compressive strengths of both beech and birch in longitudinal (*f*_*cL*_) and radial directions (*f*_*cR*_) (for normal and inert atmosphere) are listed in Table 2 (and shown in Figure S9 and Figure S10 of the supplementary material). While the *f*_*cL*_ of beech increased by 16% at moderate temperatures and then declined at more intensive treatments, especially in a nitrogen atmosphere, the *f*_*cL*_ of birch was not negatively impacted when subjected to the full range of modifications.

**Table 2:** Mean strength values (MPa) and their standard deviation % (SD) for heat-treated and untreated beech and birch by quasi-static tests (the first number refers to the treatment temperature and the second number refers to the duration time in minutes).

Beech	Ref	Air						Nitrogen					
		150–10	200–10	250–10	150–30	200–30	250–30	150–10	200–10	250–10	150–30	200–30	250–30
*f*_*cL*_	80.64	75.27	79.00	76.67	85.48	93.90	58.98	90.42	92.05	78.29	86.37	93.73	32.51
SD	7.6	6.1	9.7	16.2	6.1	7.9	13.9	8.6	3.7	15.3	6.8	10.0	28.4
*f*_*cR*_	22.64	15.96	17.68	14.11	16.97	17.13		17.12	18.28	13.77	16.98	18.90	8.18
SD	0.5	0.4	0.5	0.7	0.2	0.8		0.5	0.5	2.7	1.7	0.4	2.5
*f*_*tL*_	160.58	196.99	169.1	87.40	192.04	139.30	47.89						
SD	23.0	12.8	10.9	8.0	28.5	9.1	9.4						
*τ*_*LR*_	17.94	22.72	22.12	14.12	23.89	21.98	3.63	22.56	27.32		25.30	18.4	
SD	1.9	1.6	1.7	1.4	2.9	1.6	1.8	2	3		2.7	2.4	
Birch													
*f*_*cL*_	61.12	58.69	83.51	77.73	71.79	74.21	61.28	63.59	74.99	70.96	73.16	77.36	62.32
SD	1.1	2.9	1.9	2.2	1.7	2.4	5.4	2.0	2.1	4.0	1.0	7.7	7.2
*f*_*cR*_	9.57	10.61	10.8	10.5	9.8	9.9		10.41	10.9	8.8	11.08	11.7	4.7
SD	0.3	0.6	0.4	1	0.4	1.6		0.4	0.6	2.1	0.9	0.4	1.4
*f*_*tL*_	165.74	186.82	192.05	69.77	189.64	165.65	46.9						
SD	12.6	20.6	33.1	19.7	25.0	16.2	10.4						
*τ*_*LR*_	20.17	23.79	19.55	11.47	23.69	21.64		23.17	23.14		25.05	17.08	
SD	1.5	1.4	1.5	1.5	1.5	1.4		0.4	2.7		2.3	2.6	

The difference between the two species is even more pronounced in the radial direction; this may be explained by the effect of the wood rays, which, in beech, unlike birch, are very wide and have a significant influence on the mechanical properties. The *f*_*cR*_ of beech was adversely affected at all temperatures, whereas the *f*_*cR*_ of birch exhibited a modest increase at low temperatures, followed by a reduction at higher levels.

**Figure 5:**
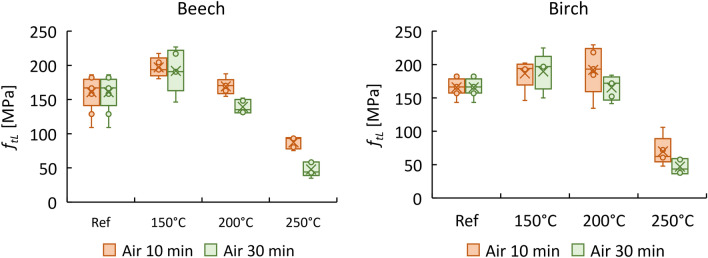
Longitudinal tensile strength (*f*_*tL*_) of beech and birch.

Corresponding to the tensile strength (*f*_*tL*_) of modified samples in the longitudinal direction, overall, birch exhibited rather similar behaviour to beech, with an increase of 23% and 16% for beech and birch, respectively, compared to *f*_*tL*_ in the standard conditions (see Fig. 5). At 250 °C, both species had lower *f*_*tL*_ values in comparison to untreated samples. Similarly, the shear strength of wood initially increased upon thermal treatment, then deteriorated at 250 °C exposure conditions, as shown in Fig. 5. In contrast to compressive and tensile strengths, the shear strength of beech presented a better response to heating (Figure S11 of the supplementary material).

### Young’s moduli, shear moduli and Poisson’s ratios results

The effect of heat treatments on different stiffnesses of beech and birch obtained from quasi-static tests is shown in Table 3 (and illustrated in Figures S12–S15 of the supplementary material, except for the tensile moduli of elasticity, which are shown here in Fig. 5).

**Table 3:** Mean values of elastic constants (GPa) and standard deviation % (SD) for heat-treated and untreated beech and birch by quasi-static tests.

Beech	Ref	Air						Nitrogen					
		150–10	200–10	250–10	150–30	200–30	250–30	150–10	200–10	250–10	150–30	200–30	250–30
*E*_*cL*_	15.35	13.7	14.06	12	14.17	14.04	9.46	15.72	18.81	4.58	15.8	14.55	2.52
SD	2.5	3.4	3.8	3.8	1.6	2	3.6	5.4	2	1.7	1.6	3.7	2.3
*E*_*cR*_	2.34	1.58	2.01	1.81	1.45	1.79		2.01	2.04	1	2.01	2.04	0.97
SD	0.06	0.23	0.21	0.2	0.13	0.21		0.2	0.17	0.25	0.16	0.19	0.16
*E*_*tL*_	17.07	16.64	17.23	16.9	16.42	16.24	15.79						
SD	2.2	1.2	1.3	1.3	1.1	0.6	2.1						
*G*_*LR*_	1.78	1.47	1.45	1.19	1.56	1.54	0.57	2.2	1.61		1.74	1.86	
SD	0.2	0.2	0.1	0.1	0.4	0.1	0.1	0.33	0.12		0.28	0.24	
*v*_*LR*_	0.47	0.357	0.411	0.472	0.439	0.395							
SD	0.07	0.01	0.02	0.08	0.03	0.00							
Birch													
*E*_*cL*_	13.23	11.64	15.92	13.68	13.38	12.28	10.43	11.04	16.19	14.13	11.79	13.54	7.77
SD	0.4	0.5	0.5	0.4	1.4	1.6	0.9	1.5	1.2	1	2.2	1.3	2.4
*E*_*cR*_	0.82	1.03	0.97	0.98	0.92	1.00		1.15	1.1	0.56	1.03	1.13	0.46
SD	0.12	0.06	0.29	0.09	0.04	0.05		0.04	0.05	0.13	0.14	0.06	0.46
*E*_*tL*_	14.75	16.56	17.34	17.26	14.51	16.59	16.88						
SD	3.02	3.19	3.53	3.55	0.27	2.18	2.93						
*G*_*LR*_	1.22	1.16	1.15	1.07	1.3	1.37		1.61	1.4		1.51	1.29	
SD	0.15	0.16	0.14	0.2	0.2	0.21		0.35	0.24		0.32	0.21	
*v*_*LR*_	0.508	0.469	0.535	0.693	0.507	0.469							
SD	0.06	0.00	0.05	0.05	0.01	0.02							

Regarding the compressive modulus of elasticity in the longitudinal direction (*E*_*cL*_), apart from the *E*_*cL*_ of birch treated at 10 min, which demonstrated an increase of around 22% at 200 °C followed by a moderate decrease, there was no noticeable difference in the *E*_*cL*_ of treated beech and birch compared to those of reference samples up to 200 °C.

The compressive moduli of elasticity in the radial direction (*E*_*cR*_) of beech and birch followed the same trend as their strength counterparts. The *E*_*cR*_ of beech decreased under all treatment conditions, while that of birch improved slightly, up to 200 °C, before declining at higher treatment temperatures (Figure S13).

**Figure 6:**
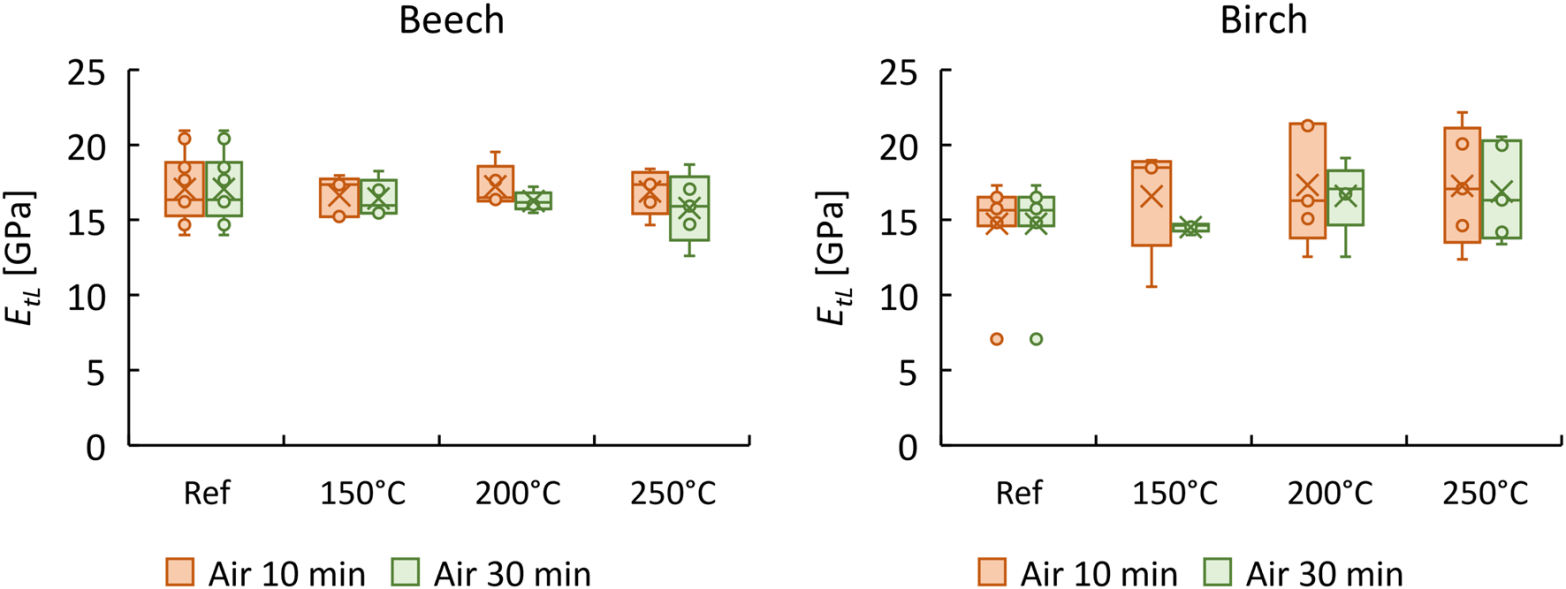
Tensile modulus of elasticity (*E*_*tL*_) of beech and birch.

Interestingly, the modulus of elasticity in tension (*E*_*tL*_) was not adversely affected by thermal treatment up to 250 °C. The* E*_*tL*_ of beech remained almost constant, while that of birch even showed an improvement in the range of 12–18% after modification, as shown in Fig. 6.

According to the shear moduli results (Table 3 and Figure S14), the heat treatments had a rather similar influence on beech and birch in air and nitrogen atmospheres, producing an increase at low temperatures followed by a decline.

Poisson’s ratio (*v*_*LR*_) for treated beech and birch demonstrated different trends from the shear moduli. The *v*_*LR*_ of beech decreased slightly with temperatures up to 200 °C, then returned to the level of untreated samples, whereas the *v*_*LR*_ of birch continued to increase with temperature (Figure S15).

### Quasi-static tests discussion

Overall, measured strength and stiffness demonstrated mild improvement under moderate temperature treatment, before falling because of the complete loss of the material bearing capacity. This general behaviour of beech and birch after thermal modification is consistent with literature findings for different wood species^17,20,32,33^. The experimental results indicated that thermal modification up to 200 °C did not result in pronounced destructive effects of the chemical compounds. There were increased condensation reactions of lignin, which meant that, in general, the mechanical properties tended to improve. As a consequence, increased elastic moduli and enhanced natural durability were expected.

With higher temperatures, the degradation of the chemical structure of wood and the decrease in its properties become more prominent, leading eventually to a complete loss of the material bearing capacity. Careful examination of the results revealed that there was a more significant decrease in the material performance of beech compared to birch wood, occurring at earlier stages of modifications. This is in line with the findings of Borůvka et al.^17^ and can be attributed to the different chemical composition of the two species, namely mannan fractions of hemicelluloses. Mannans, whose percentage is higher in birch, are more skeleton than filling material, having good links with the cellulose and more mechanically resistant to thermal degradation.

It is worth to note that the compressive strength and stiffness of radially oriented beech do not follow the general trend of improvement at moderate heating, but rather show a continuous decline with temperature, as mentioned in the previous section. This inferior behaviour of beech compared to that of birch along the radial direction is, as already mentioned, probably linked to their ray cells content. Parenchyma cells are more influenced by the heat treatment and are present in a higher percentage in beech wood^34^, negatively impacting the mechanical response of heat-treated beech.

Furthermore, the properties of hemicelluloses and lignin are reported to have more pronounced effect in the transverse direction^35^. Hence, the less thermally stable components of hemicellulose in beech are expected to have a more noticeable effect on its radial resistance.

The results of this study reveal that Young’s moduli obtained from tension were higher than those from compression tests for reference and thermally treated beech and birch. This trend is similar to that obtained by Wetzig et al.^36^, where greater *E*_*L*_ values from tensile than compression tests were obtained for heat-treated and untreated beech. This tension–compression asymmetry of wood elastic properties was also noticed for untreated beech^37^ and for untreated walnut and cherry wood^38,39^. Higher ratios than 1 for tensile to compressive stiffness were noticed when performing tensile and compression tests on the same sample sets to minimise data scattering resulting from wood heterogeneity.

The obtained shear stiffnesses values for reference and heat-treated beech were higher than values reported in the literature (e.g. Gomez-Royuela et al.^20^). It should be emphasised that the shear parameters in that study were obtained from compression tests and a direct comparison between the results may not be completely feasible, as the test method itself can have a considerable influence on the results. The shear stiffness values for reference birch agree well with data reported in the literature^40^. To the best of the authors’ knowledge, no shear moduli of heat-treated birch have been reported in literature (not even from ultrasonic tests).

Poisson’s ratio (*v*_*LR*_) values reported in literature seem to be insensitive to thermal treatment, with no clear correlations between these values and intensity of treatment^20,36^. In contrast, *v*_*LR*_ values of treated samples obtained in this study appeared to have lower values compared to untreated beech and higher values compared to reference birch. Notably, the radial deformation increased when the last treatment was performed (250 °C–10 min). However, it should be emphasised that the number of samples used in this test was limited and a deeper investigation on a higher number of specimens should be carried out for a better understanding of the contraction of the cross section in thermally treated wood. A possible reason could be that the treatment also leads to small cracks, which allows higher deformation to be observed during quasi-static testing, even though the material has become stiffer.

**Figure 7:**
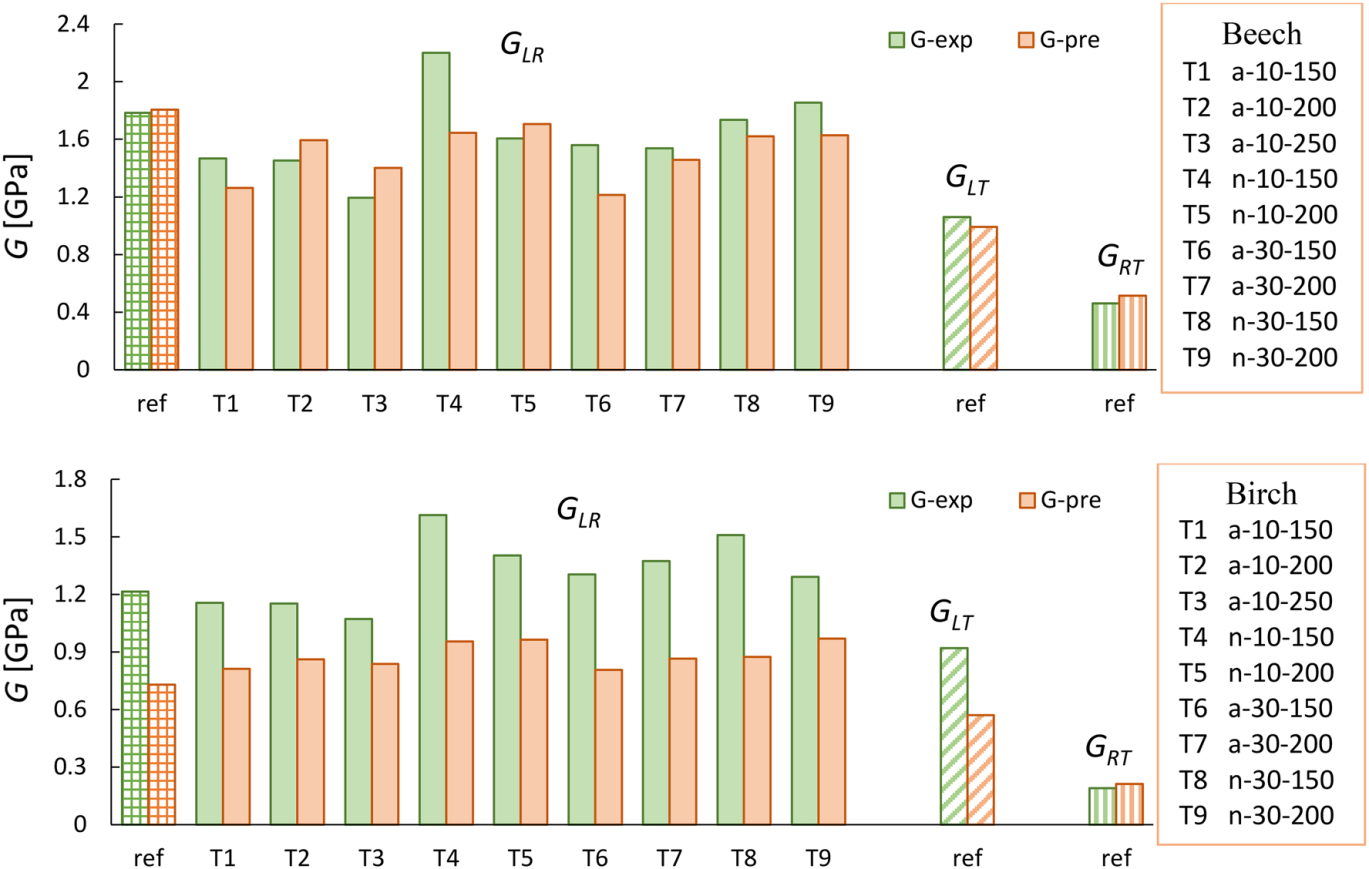
A comparison between experimental shear modulus (G-exp) with predicted values (G-pre) using the Saint-Venant model for beech and birch (a: air condition; n: nitrogen condition).

Now, by using Young’s moduli and Poisson’s ratios of thermally treated and untreated beech and birch obtained in this study (and elastic constant values from literature for *E*_*T*_, *v*_*LT*_ and *v*_*RT*_), it is possible to calculate *G* for beech and birch using the Saint-Venant relation^21^ given in Eq. 1. The results are shown in Fig. 7.

As illustrated, the predicted shear moduli agree well with measured values, with approximately 11.7% and 25% error for untreated and treated beech, respectively, which is well within the range of the natural variability of wood. Meanwhile, the predicted *G* values for birch were less accurate, with 40% and 42% lower values attained for untreated and treated samples.

**Figure 8:**
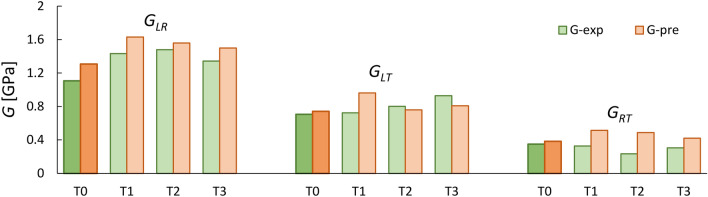
A comparison between experimental shear modulus (reported in Gòmez-Royuela et al. ^20^) with predicted values using the Saint-Venant model for untreated and modified beech.

To further confirm the validity of the Saint-Venant principle for predicting shear values of thermally treated beech, the data reported in Gomez-Royuela et al.^20^ were used here to estimate the shear moduli of beech; the results are shown in Fig. 8. For more details about the thermal treatment used, the reader is referred to the published study. The predicted *G* values were 5–18% lower than the experimental values for reference samples. However, this relation overestimated *G*_*RT*_ values of modified beech by up to 108%.

## Ultrasonic characterisation: results and discussion

The results are grouped into Young’s moduli, shear moduli and Poisson’s ratios. A comparison with their corresponding obtained parameters from quasi static tests (discussed earlier) is made where possible. For other parameters, elastic constants for unmodified beech and birch reported in Hearmon^40^ are presented instead.

### Young’s moduli

**Figure 9:**
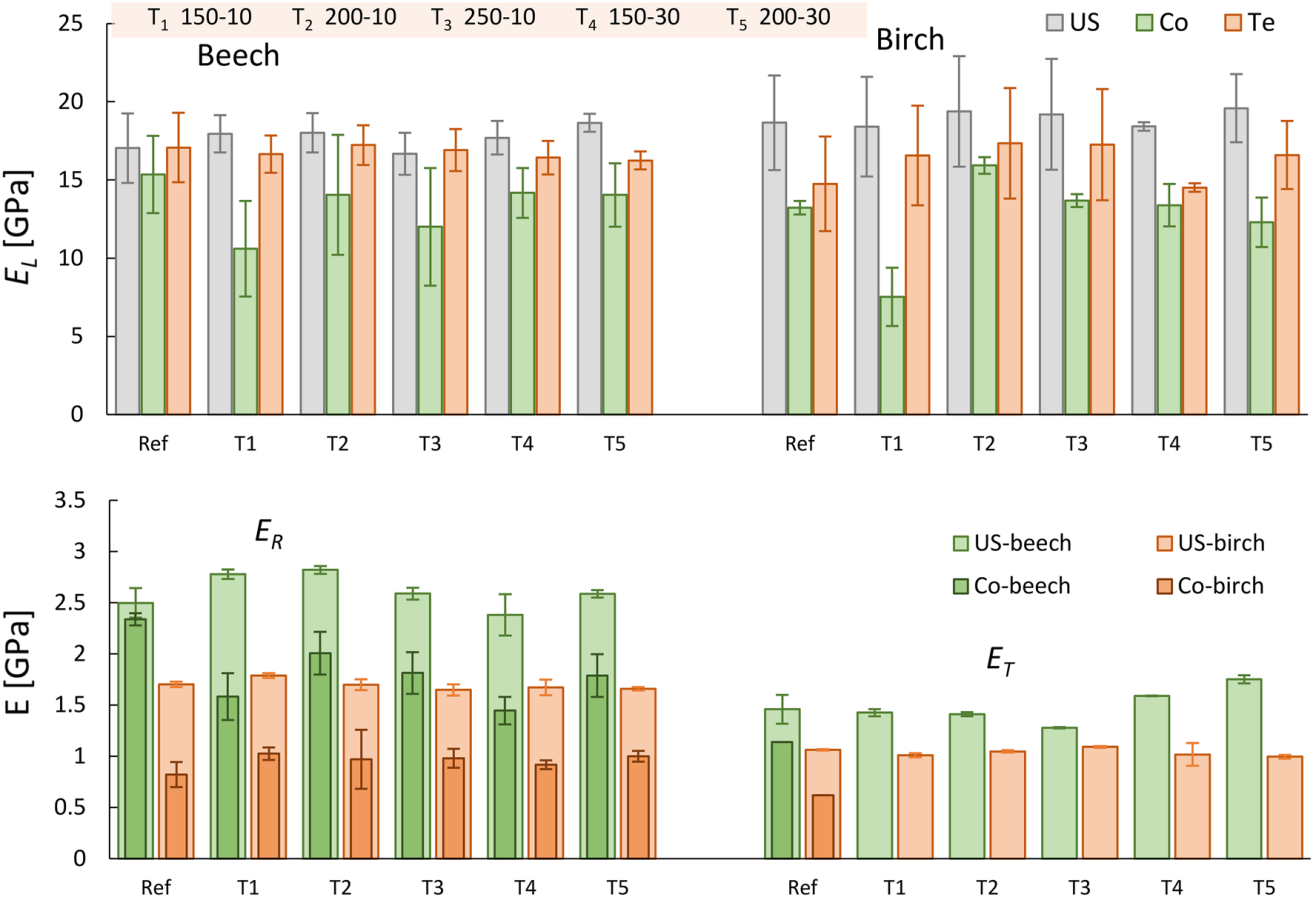
Mean values for the Young’s moduli of reference and heat-treated beech and birch obtained by ultrasound (US) and quasi-static tests (Co: compression; Te: tension).

Figure 9 shows the mean values and ranges of variation of Young’s moduli obtained from different heat treatments using ultrasonic waves and quasi-static testing for beech and birch samples.

Overall, the Young’s moduli from ultrasonic tests have higher values than those obtained from quasi-static tests in all directions. This general tendency is well documented in the literature^36,41,42^. This difference, however, is more noticeable in the radial direction (up to 75% and 107% higher values obtained by ultrasound for beech and birch, respectively). Gomez-Royuela et al.^20^ reported 62% higher values of *E*_*R*_ obtained by ultrasound compared to values from compression tests for heat-treated beech. Interestingly, the ultrasound *E*_*R*_ of beech did not seem to be affected by different thermal treatments, with values up to 13% higher than reference samples. This was not observed for *E*_*R*_ from quasi-static tests.

It can be seen from the results that ultrasonic stiffnesses are in closer agreement with their corresponding tensile stiffnesses rather than with their compressive stiffnesses. The highest measured difference was around 14.8% and 26.9% compared to 69% and 59% when considering tension and compression for beech and birch, respectively. This trend can also be observed from the data reported in Bachtiar et al.^38^ for walnut wood, where a difference of less than 2% was found for *E*_*L*_ and *E*_*R*_ in tension compared to ultrasound.

It should be emphasised here that the elastic modulus was calculated from the elastic part of the loading path, rather than from the unloading path of the stress–strain curve. Akter^43^ found that the unloading stiffness was about 7–30% higher than the loading stiffness when testing spruce specimens in tangential and radial directions, regardless of whether tensile or compressive loading was applied. Therefore, if the unloading stiffness is used, there will probably be a closer resemblance between stiffness values measured from ultrasound and quasi-static tests.

It is not uncommon in the literature to use the simplified method for calculating Young’s moduli of wood (*E*_*i*_ ≈ *C*_*ii*_ = *ρ*.*V*_*ii*_^2^) from ultrasounds (e.g. Wetzig et al.^36^). Now, inspection of the *C* values for reference beech and birch (listed in Table S2 in the supplementary material; *C*_*11(beech)*_ = 23.7; *C*_*11(birch)*_ = 21.4 GPa) clearly shows that the simplified analysis method overestimates the Young’s moduli of wood and it is therefore strongly recommended to use the full stiffness inversion method when determining the elastic modulus from ultrasonic tests.

### Shear moduli

**Figure 10:**
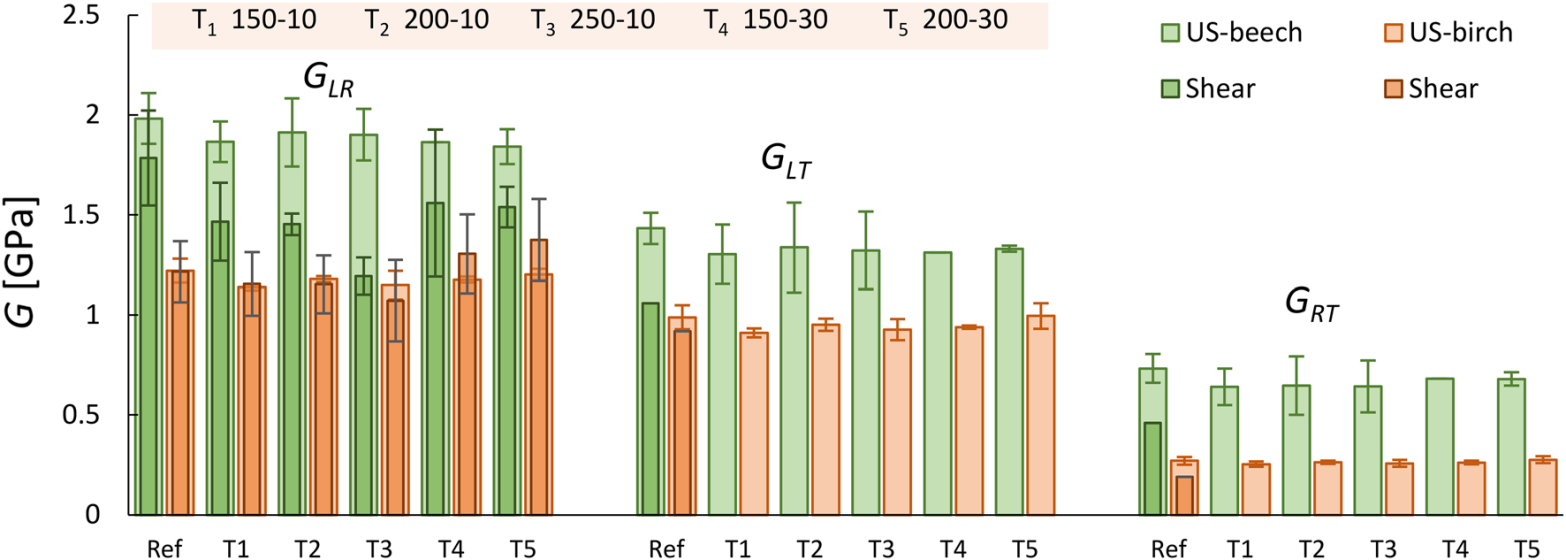
Mean values of the shear moduli of reference and heat-treated beech and birch obtained by ultrasound (US) and quasi-static tests (Co: compression; Te: tension).

The shear moduli obtained from ultrasound tests are illustrated in Fig. 10. While ultrasound overestimated the *G*s of beech by approximately 11–59%, the ultrasonic *G*s of birch agreed well with those obtained from quasi-static tests. In general, the ultrasonic results of beech followed the same trend as quasi-static tests, in which similar or even lower shear modulus values were obtained after thermal treatment. Unlike Young’s moduli, *G* parameters are independent of *ν* and can therefore be easily measured using one geometry, as the off-diagonal terms of the stiffness matrix are not needed.

### Poisson’s ratios

**Figure 11:**
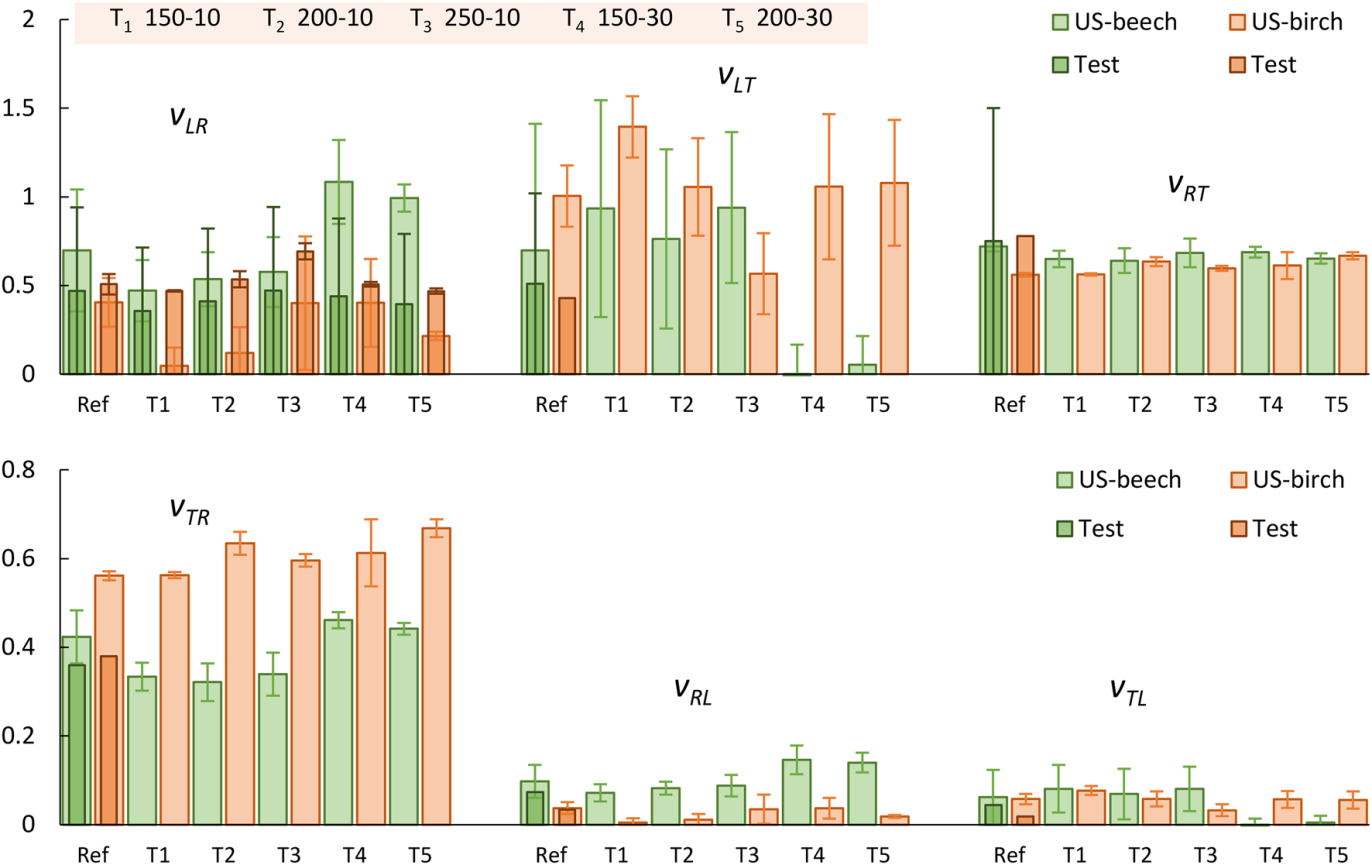
Mean values for the Poisson’s ratios of reference and heat-treated beech & birch obtained by ultrasound and quasi-static tests.

The six Poisson’s ratios of beech and birch obtained from ultrasonic tests are presented in Fig. 11. As shown, Poisson’s ratios from ultrasound tests were reasonably comparable to those from quasi-static tests for untreated beech and birch, but not for thermally treated samples, for which values (for* ν*_*LR*_) exceeding 1 can be noticed. To the authors’ knowledge, there are no reported values for Poisson’s ratios in heat-treated birch, which prevents comparison for the other Poisson’s ratio results.

Unlike quasi-static tests, there is no clear correlation between Poisson’s ratios and heat treatment intensity from ultrasonic tests, which corresponds well with observations from the literature for heat-treated beech^20^. Overall, the Poisson’s ratio values obtained in the present research were of the same order of magnitude as those measured using ultrasounds in the study of Gomez-Royuela et al.^20^, except in some cases, namely, *ν*_*LT*_ and *v*_*TL*_, in which negative values and values above 1 were obtained (see Fig. 11). While such values are reported in the literature for different wood species^30,31,38,44,45^ and wood-based composites^46^, they are still deemed unusual for wood and wood composites.

To assess these results, we have to keep in mind how the ultrasonic tests on the cubic samples were performed: For each set of test parameters, there are four test repetitions, making use of all orientations of the cubes which (theoretically) should render identical results. The mean standard deviation of these test repetitions is about 0.6%. Yet, to find all terms of the stiffness matrix, four cubes of each type with different orientations, as shown in Fig. 3, are needed. Therefore, each local difference in the material itself as well as each deviation from the ideal orientation of the axes will result in increased errors in the calculated terms of the stiffness matrices. Given the nature of the wood material, to a certain degree such errors are inevitable. This being so, the mean values for the moduli, based on the ultrasonic tests, can be considered acceptable: For beech wood, the mean value is about 9% of the calculated terms for *E* and *G*, while for birch wood it is about 3%. For Poisson's ratios, the mean values are higher, though (24% and 16%). To increase the reliability and the precision of the results, it would be necessary to increase the number of tested samples.

## Conclusions

Overall, the obtained strength and stiffness for beech and birch exhibited mild improvement under moderate temperature treatment, followed by a decrease as a result of the loss of the material bearing capacity. This improvement was slightly more noticeable under nitrogen treatment than in air. Nevertheless, a more pronounced decrease in the material properties of beech in comparison to birch was found, occurring at earlier stages of modifications. This can be attributed to the different chemical composition of the two species.

Unlike beech properties in the longitudinal direction, radially oriented beech samples rather demonstrated a continuous decline with temperature, which is probably due to their higher ray cells content compared to birch.

In this study, higher Young’s moduli were obtained from tensile than from compression tests for reference and thermally treated beech and birch, confirming the tension–compression asymmetry of beech and birch.

As for shear strength and moduli results, the heat treatments had a rather similar effect on beech and birch in air and in a nitrogen atmosphere, namely an increase at low temperatures followed by a decline.

In contrast to findings in the literature, the *v*_*LR*_ values of treated samples detected in this study seemed to have lower values compared to untreated beech and higher values compared to untreated birch. Notably, the radial deformation increased when treatment at 250 °C–10 min was performed.

The Saint-Venant model was able to predict the shear moduli of untreated and treated beech with approximately 11.7% and 25% error, respectively, which is well within the range of the natural variability of wood. Meanwhile, the model underestimated the *G* values of birch, with 40% and 42% lower values obtained for untreated and treated samples.

The ultrasonic stiffnesses obtained were in a closer agreement with their corresponding tensile stiffnesses than their compressive stiffnesses in the longitudinal orientation. The highest measured difference was around 14.8% and 26.9% compared to 69% and 59% for tension and compression of beech and birch, respectively. This difference, however, was more prominent in the radial direction (up to 75% and 107% higher values obtained by ultrasounds compared to compression tests for beech and birch, respectively).

The shear moduli obtained from ultrasound tests for birch agreed well with those obtained from quasi-static tests, whereas those for beech overestimated the *G*s by approximately 11–59% compared to quasi-static tests.

Interestingly, Poisson’s ratios from ultrasound tests were reasonably comparable with those from quasi-static tests for untreated beech and birch, but not for thermally modified samples, for which negative values and exceeding 1 values can be observed for *ν*_*LT*_ and *v*_*TL*_. To increase the reliability and the precision of the results, it would be necessary to increase the number of tested samples. Yet, the results given so far in this paper can give directions for future choices of test parameters and/ or test objects.

## Supplementary Information

Supplementary Information.

## Data Availability

The data used to support the findings of this study are available from the corresponding author upon request.
