# Supplementary material for: Characterisation of thermally treated beech and birch by means of quasi-static tests and ultrasonic waves
Source: Sci Rep. 2023 Apr 18;13:6348. doi: 10.1038/s41598-023-33054-w (PMC10113205; doi:10.1038/s41598-023-33054-w)
Supplement: Supplementary file 1 — Supplementary Information. [file 41598_2023_33054_MOESM1_ESM.docx]

**Supplementary material for Characterisation of thermally treated beech and birch by means of quasi-static tests and ultrasonic waves**

**Physical properties results and discussion**

A darkening in the colour of different samples was observed depending on the level of heat treatment; the higher the temperature, the more intense the colour change (see Fig. S-1-S-6). This effect is well documented in literature for different wood species [1-5]. In fact, Pleschberger et al. [3] found a strong correlation between measured fracture and colour values for thermally treated spruce and ash woods, suggesting the possibility of using colour parameters in creating a non-destructive quality control for strength grading of TT wood.

Figure S7 shows mass loss (ML) and density loss (DL) of treated beech and birch samples. As illustrated, there are no substantial differences between the response of the two wood species and also between the air and nitrogen treatment. There is only a small degradation of the material up to 200°C treatment. Beyond this temperature, the change is more pronounced with a mass loss higher than 30% and a density loss in the range of 15 to 20% at 250 °C - 30 minutes treatment.

The equilibrium moisture contents of the modified material are presented in Figure S8. The reported mean values are calculated considering all geometries for each treatment condition. Again, no meaningful difference between the moisture content trend of modified beech and birch can be seen. EMC Gradually decreases with the increasing treatment severity and reaches to approximately 6.8 - 4.2% at the highest treatment temperature. Different mechanisms are suggested in literature to explain the change in EMC after thermal treatment. The reduction of the content of hydroxyl groups, the primary sorption sites, in the cell wall polymer matrix [6, 7], the enhanced inaccessibility of hydroxyl groups to water molecules as a result of increased cellulose crystallinity [8, 9] and cross-linking reactions of lignin [9, 10] are among the most discussed reasons for EMC reduction. In any case, this reduction in EMC is expected to improve the dimensional stability of modified beech and birch, rendering them suitable for automotive applications as discussed in the introduction section.


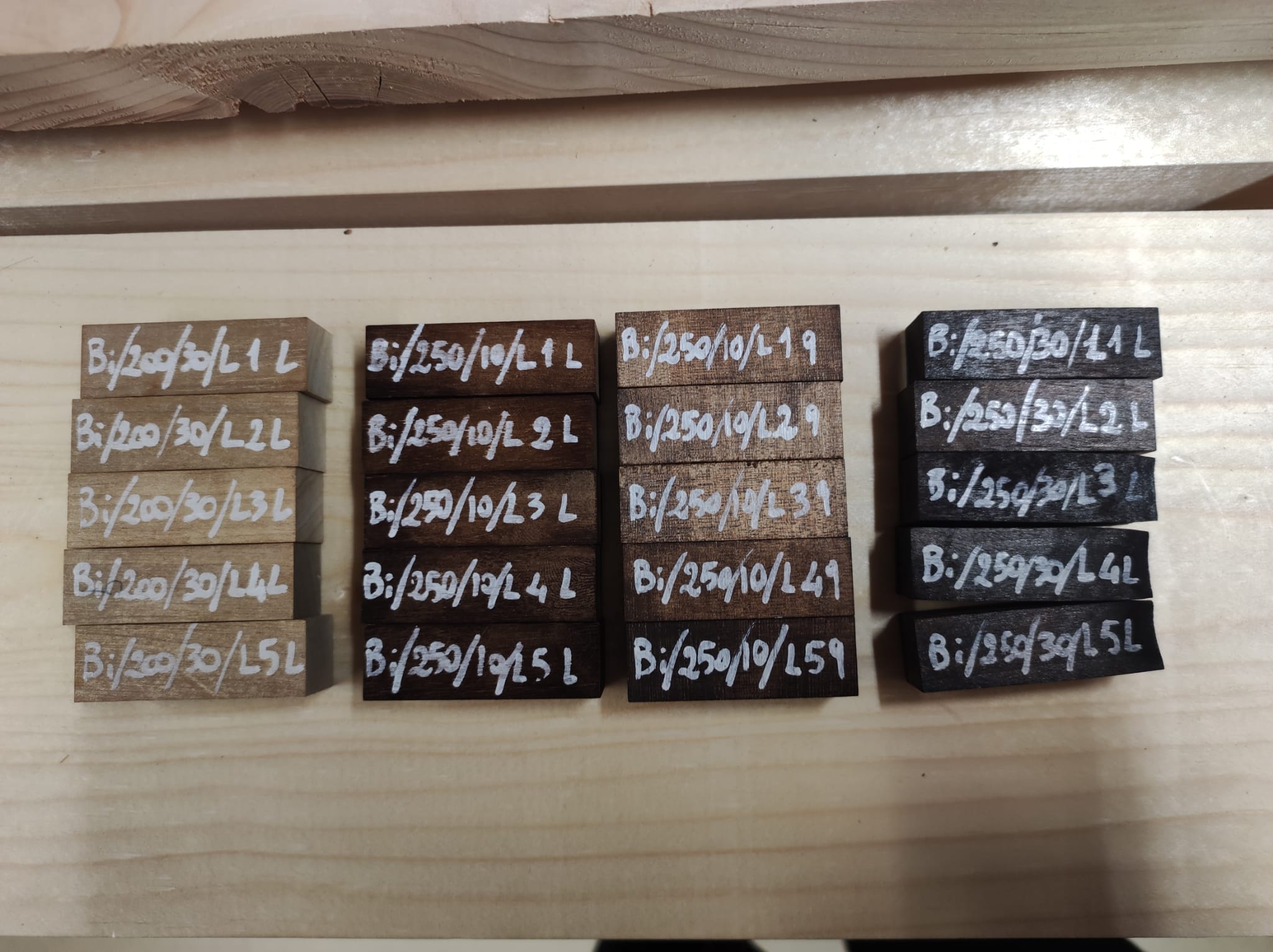


**Figure S1.** Treated compression sample: longitudinal orientation of beech and birch


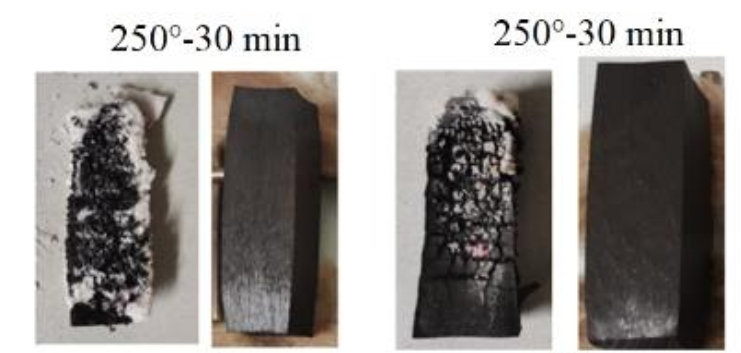


a

b

**Figure S2.** Treated compression sample: radial orientation (compared to longitudinal) of (a) beech and (b) birch in air condition.


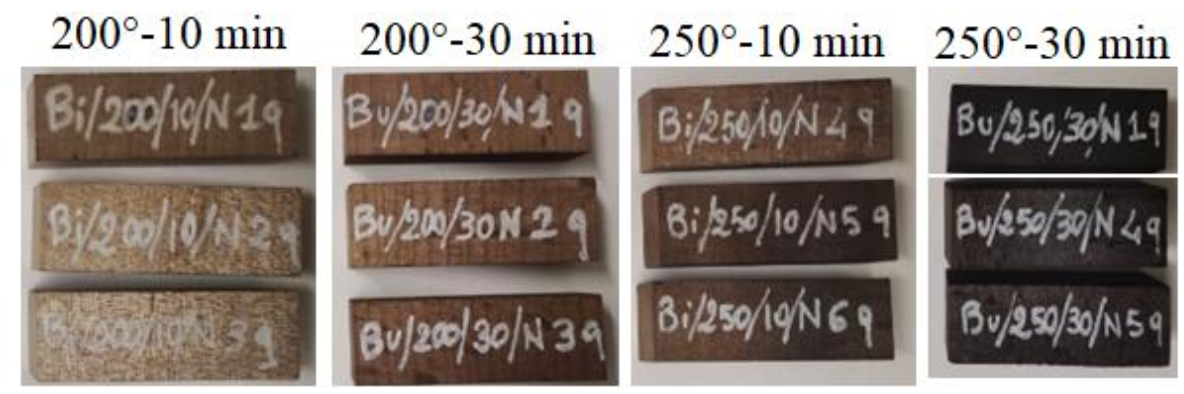


**Figure S3.** Treated compression samples, radial orientation, nitrogen atmosphere.


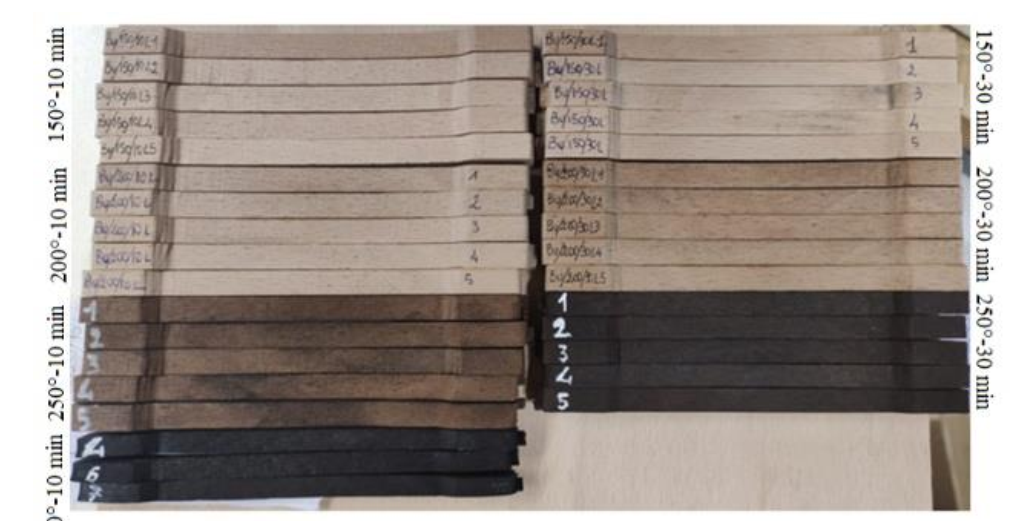


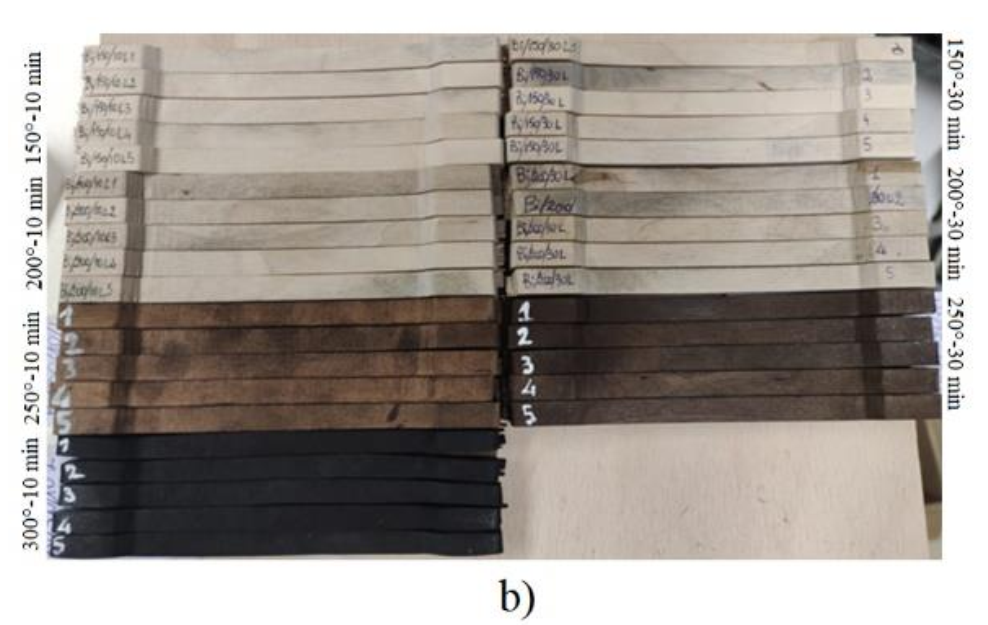


**Figure S4.** Beech (a) and birch (b) treated tensile samples, air condition.


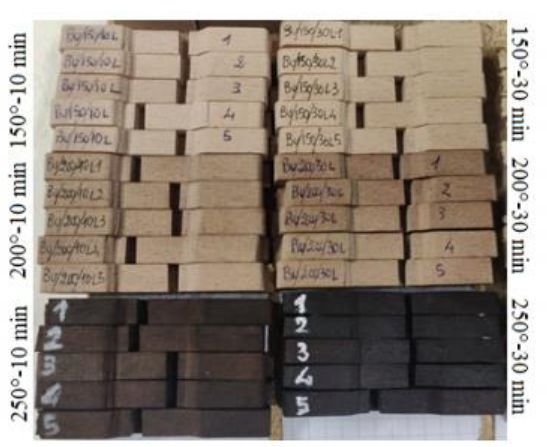

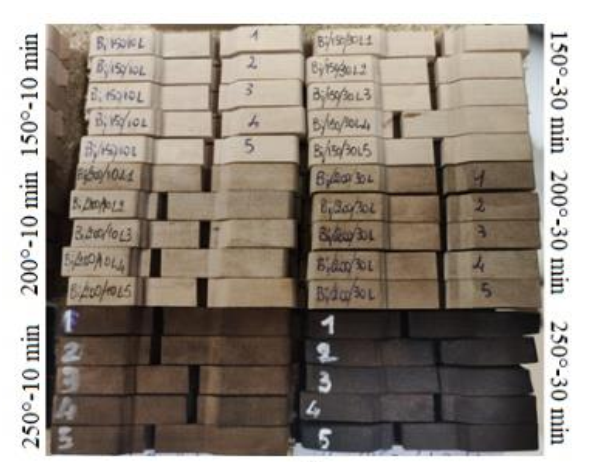

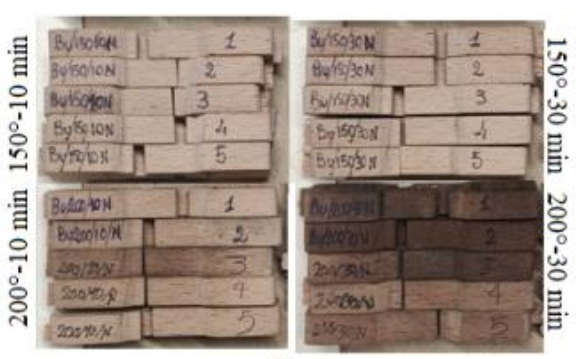

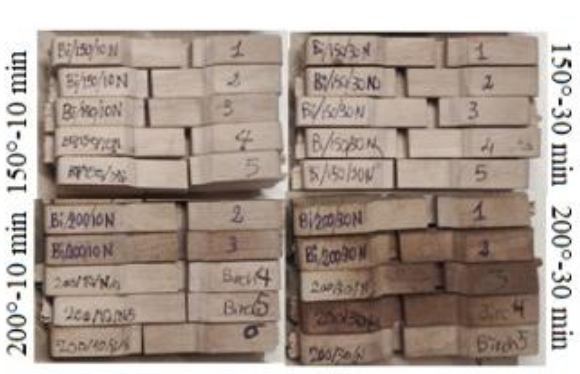


(a)

(c)

(d)

(b)

**Figure S5.** Treated shear sample: beech, in air (a) and nitrogen condition (b); birch in air (c) and nitrogen.


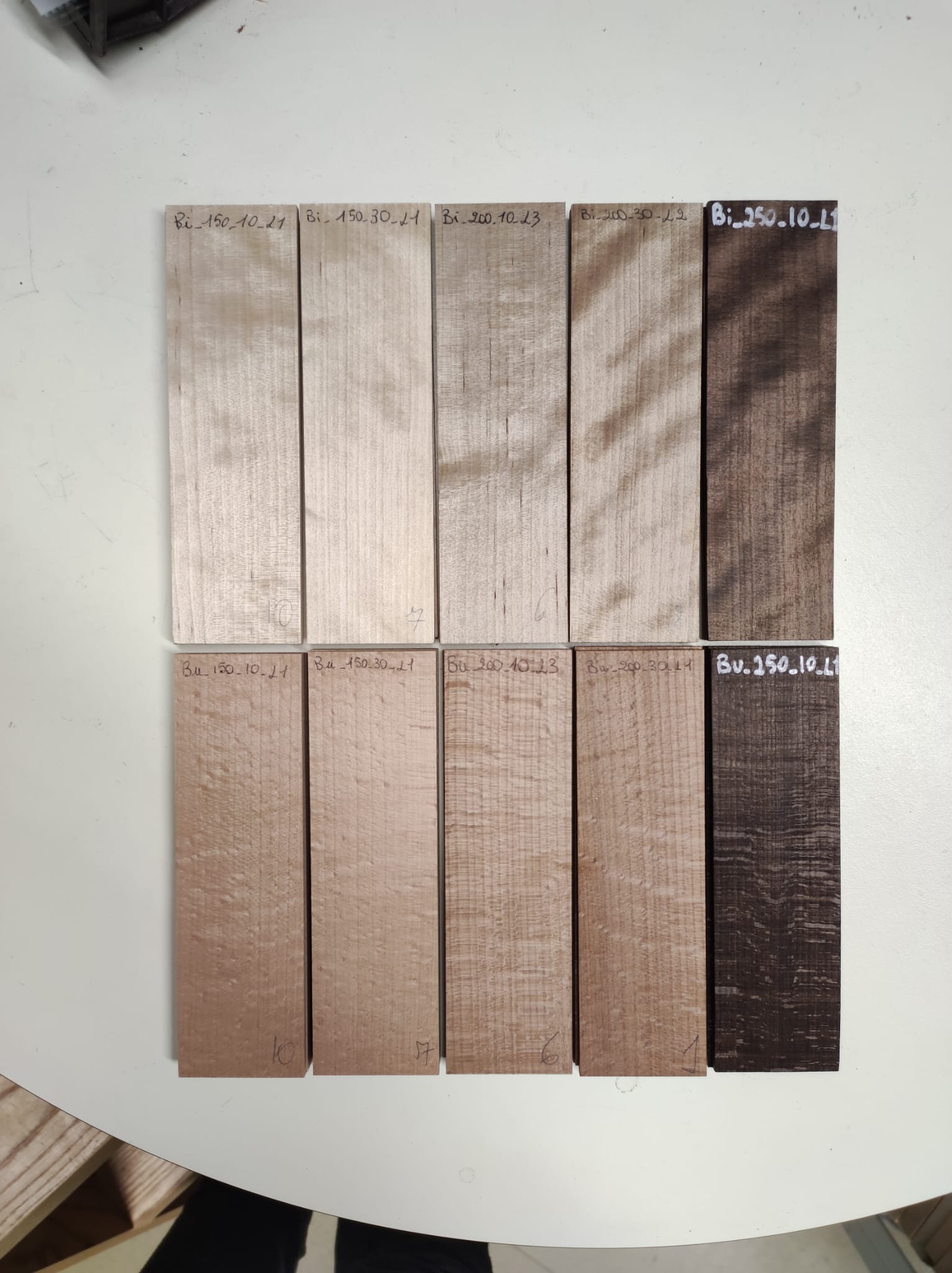


**Figure S6.** Treated Poisson’s ratio samples, longitudinal orientation, air atmosphere.

**Figure S7.** Percentage of mass loss (ML) and density loss (DL) in treated beech and birch.

**Figure S8.** Equilibrium moisture content in % (EMC) of beech and birch.

Table S1. Treatment conditions that are statistically significant (p < 0.05) than their reference conditions for both beech and birch (the first number represents temperature [°C], the second number represents treatment time [min] while A stands for air and N stands for nitrogen).

| Property | Beech | | | Birch | | |
| --- | --- | --- | --- | --- | --- | --- |
|  | B^(1)^ | G-H^(2)^ | M-W U^(3)^ | B^(1)^ | G-H^(2)^ | M-W U^(3)^ |
| *f_cL_* | ___ | ___ | 250-30-A  250-30-N |  | 200-10-A  250-10-A  150-30-A  200-30-A  250-30-A  150-10-N  200-10-N  250-10-N  150-30-N | 200-10-A  250-10-A  150-30-A  200-30-A  250-30-A  150-10-N  200-10-N  250-10-N  150-30-N  200-30-N |
| *f_cR_* |  | 150-10-A  200-10-A  250-10-A  150-30-A  200-30-A  250-30-A  150-10-N  200-10-N  250-10-N  150-30-N  200-30-N  250-30-N | 150-10-A  200-10-A  250-10-A  150-30-A  200-30-A  250-30-A  150-10-N  200-10-N  250-10-N  150-30-N  200-30-N  250-30-N |  | 200-10-A  200-30-N  250-30-N | 200-10-A  200-10-N  200-30-N  250-30-N |
| *f_tL_* | 150-10-A  250-10-A  250-30-A |  | 150-10-A  250-10-A  250-30-A | 250-10-A  250-30-A |  | 250-10-A  250-30-A |
| *τ_LR_* | 150-30-A  250-30-A  200-10-N  150-30-N |  | 150-30-A  250-30-A  200-10-N  150-30-N |  | 250-10-A | 250-10-A |
| *E_cL_* |  | 250-10-N  250-30-N | 250-10-N  250-30-N |  | 150-10-A  200-10-A  250-30-A | 150-10-A  200-10-A  250-30-A |
| *E_cR_* | 150-10-A  250-10-A  150-30-A  200-30-A  250-10-N  250-30-N |  | 150-10-A  250-10-A  150-30-A  200-30-A  250-10-N  250-30-N | 150-10-N  200-10-N  250-10-N  200-30-N  250-30-N |  | 150-10-N  200-10-N  250-10-N  200-30-N  250-30-N |
| *E_tL_* |  |  |  |  |  |  |
| *G_LR_* | 250-10-A  250-30-A |  | 250-10-A  250-30-A |  |  |  |

^(1)^ Bonferroni; ^(2)^ Games-Howell; ^(3)^ Mann-Whitney U test.

**Quasi-static testing results**

In figures S9 to S15, A box and whisker chart shows distribution of data into quartiles, highlighting the mean and outliers. The lines that extend vertically are called “whiskers”. These lines indicate variability outside the upper and lower quartiles, and any point outside those lines or whiskers is considered an outlier, plotted as a circle. The mean markers are displayed as a cross.


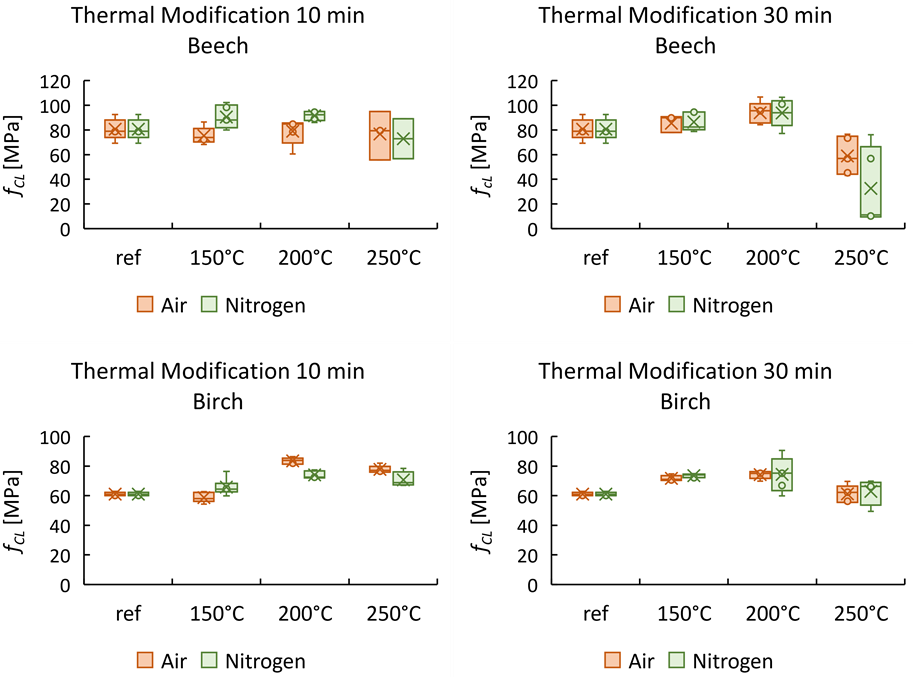


**Figure S9.** Longitudinal compressive strength (*fc_L_*) of beech and birch.

**Figure S10.** Radial compressive strength (*fc_R_*) of beech and birch.

**Figure S11.** Shear strength related to the longitudinal-radial plane (*τ_LR_*) of beech and birch.

**Figure S12.** Compressive modulus of elasticity in longitudinal direction (*E_cL_*) of beech and birch.

**Figure S13.** Compressive modulus of elasticity in radial direction (*E_cR_*) of beech and birch

**Figure S14.** Shear modulus of elasticity (*G_LR_*) of beech and birch.

**Figure S15.** Poisson’s ratio (*v_LR_*) of beech and birch.

Table S2: *C_11_* values for thermally treated and untreated beech and birch.

**References**

[1] AYDEMIR, D., GUNDUZ, G. & OZDEN, S. 2012. The influence of thermal treatment on color response of wood materials. *Color Research and Application,* 37**,** 148-153.

[2] SRINIVAS, K. & PANDEY, K. K. 2012. Effect of Heat Treatment on Color Changes, Dimensional Stability, and Mechanical Properties of Wood. *Journal of Wood Chemistry and Technology,* 32**,** 304-316.

[3] PLESCHBERGER, H., TEISCHINGER, A., MÜLLER, U. & HANSMANN, C. 2014. Change in fracturing and colouring of solid spruce and ash wood after thermal modification. *W****ood Material Science & Engineering***9**,** 92-101.

[4] KUCEROVA, V., LAGANA, R., VYBOHOVA, E. & HYROGOVA, T. 2016. The Effect of Chemical Changes during Heat Treatment on the Color and Mechanical Properties of Fir Wood. *Bioresources,* 11**,** 9079-9094.

[5] Marcon, B., Goli, G., Matsuo-Ueda, M., Denaud, L., Umemura, K., Gril, J. and Kawai, S., 2018. Kinetic analysis of poplar wood properties by thermal modification in conventional oven. iForest: Biogeosciences and Forestry, 11(1), pp.131-139.

[6] JÄMSÄ, S. & VIITANIEMI, P. Heat treatment of wood–Better durability without chemicals. In: P*roceedings of special seminar held in Antibes*, 2001 France.

[7] HILL, C., ALTGEN, M. & RAUTKARI, L. 2021. Thermal modification of wood-a review: chemical changes and hygroscopicity. *Journal of Materials Science,* 56**,** 6581-6614.

[8] WIKBERG, H. & MAUNU, S. L. 2004. Characterisation of thermally modified hard- and softwoods by C-13 CPMAS NMR. *Carbohydrate Polymers,* 58**,** 461-466.

[9] BOONSTRA, M. J. & TJEERDSMA, B. 2006. Chemical analysis of heat treated softwoods. *Holz Als Roh-Und Werkstoff,* 64**,** 204-211.

[10] TJEERDSMA, B. F. & MILITZ, H. 2005. Chemical changes in hydrothermal treated wood: FTIR analysis of combined hydrothermal and dry heat-treated wood. *Holz Als Roh-Und Werkstoff,* 63**,** 102-111.
